# The association between pan-immune-inflammation value and chronic obstructive pulmonary disease: data from NHANES 1999–2018

**DOI:** 10.3389/fphys.2024.1440264

**Published:** 2024-10-07

**Authors:** Shuhui Qiu, Qi Jiang, Yang Li

**Affiliations:** Department of Respiratory and Critical Care Medicine, First Hospital of Jilin University, Changchun, Jilin, China

**Keywords:** COPD, pan-immune-inflammation value, NHANES, cross-sectional study, cohort study

## Abstract

**Background:**

The pan-immune-inflammation value (PIV) is an emerging biomarker quantitatively reflecting the systemic immune-inflammatory status. The predictive value of PIV has been well-established across various clinical settings. However, its role in chronic obstructive pulmonary disease (COPD) remains unclear and necessitates further investigation.

**Methods:**

Data from NHANES 1999–2018 were filtered. Logistic regression analyses were used to assess the correlation between COPD prevalence and PIV in all participants. COX regression analyses and Kaplan-Meier survival curves were used to investigate the relationship between COPD all-cause mortality and PIV in COPD patients. Restricted cubic spline (RCS) analyses and piecewise linear regression analyses were additionally employed to explore the correlation between PIV and COPD. Subgroup analyses were performed to further clarify the effects of other covariates on the associations. Sensitivity analyses were employed to assess the robustness of the results.

**Results:**

A total of 28,485 participants aged 40 years and older were recruited for this study. After fully adjusting for covariates, higher PIV levels were independently associated with increased COPD prevalence (OR = 1.67; 95% CI: 1.39–2.01) and all-cause mortality (HR = 2.04; 95% CI: 1.41–2.95). The COPD prevalence curve exhibited an inflection point at Log10-PIV of 2.24, showing no significant correlation on the left side (OR = 0.86; 95% CI: 0.45–1.64) but a positive correlation on the right side (OR = 2.00; 95% CI: 1.57–2.55). The COPD all-cause mortality curve displayed an inflection point at Log10-PIV of 2.38, indicating a negative correlation on the left side (HR = 0.23; 95% CI: 0.12–0.44) and a positive correlation on the right side (HR = 4.12; 95% CI: 2.62–6.48). Subgroup analyses with interaction tests showed that the strength of the correlation between PIV and COPD prevalence was influenced by race, smoking status, and BMI (all p for interaction <0.05). The relationship between PIV and COPD all-cause mortality was unaffected by any covariates (all p for interaction >0.05).

**Conclusion:**

Elevated PIV levels are associated with increased COPD prevalence. COPD patients with either elevated or reduced PIV levels experience higher all-cause mortality. Further large-scale, longitudinal studies are required to corroborate these findings.

## 1 Introduction

Chronic obstructive pulmonary disease (COPD) is a prevalent chronic airway disease characterized by persistent airway inflammation leading to irreversible airflow limitation (Christenson et al., 2022; Rao et al., 2020). According to the World Health Organization, COPD is the third leading cause of death globally, causing 3.23 million deaths in 2019 (World Health Organization, 2024). It is estimated that between 2020 and 2050, the direct and indirect costs of COPD worldwide will reach 4.326 trillion US dollars, imposing a significant burden on the global economy and society (Chen et al., 2023). Although COPD can affect individuals of all age groups, it is most common in those over 40 years old (Agustí et al., 2020). The pathogenesis of COPD is complex and heterogeneous, influenced by various factors, including environmental and genetic factors (Christenson et al., 2022). Novel and improved biomarkers for airway diseases may help address the clinical and biological complexities of COPD and facilitate precision medicine (Agusti et al., 2016). Further research is needed to discover reliable, measurable, and clinically relevant biomarkers to enhance COPD prevention and improve the prognosis of COPD patients.

The pan-immune-inflammation value (PIV), also known as the aggregate index of systemic inflammation (AISI), is a recently developed biomarker that relies on peripheral blood counts, calculated by using the counts of neutrophils, platelets, monocytes, and lymphocytes, which is believed to be indicative of systemic inflammation (Guven et al., 2022; Yang et al., 2022). Previous studies have demonstrated an association between PIV or AISI and the severity as well as clinical outcomes of numerous immune and inflammation-related diseases, including esophageal cancer (Feng et al., 2023), non-small cell lung cancer (Zhai et al., 2023), abdominal aortic calcification (Jin et al., 2024), and myocardial infarction (Murat et al., 2023). It is noteworthy that a recent study has investigated the prognostic role of AISI in COVID-19 patients with COPD(13). Large-scale studies examining the association between PIV and COPD, however, remain absent.

To address this knowledge gap, we utilized a substantial sample of individuals aged 40 and older from the National Health and Nutrition Examination Survey (NHANES) to assess the association between PIV levels and the COPD prevalence and all-cause mortality. We also employed restricted cubic spline (RCS) curves and segmented linear regression to explore their nonlinear relationships and applied subgroup analyses to identify specific populations.

## 2 Methods

### 2.1 Study design and participants

The National Health and Nutrition Examination Survey (NHANES) is a nationwide survey conducted by the National Center for Health Statistics (NCHS) under the Centers for Disease Control and Prevention to collect data on the health of the population. It has been conducted biennially since 1999. We used publicly available NHANES data from 1999 to 2018 for this study. Each NHANES survey cycle underwent rigorous evaluation and approval by the NCHS Research Ethics Review Board, and all participants provided written informed consent (CDC, 2024a). The dataset is available publicly on the NHANES website (CDC, 2024b).

**FIGURE 1:**
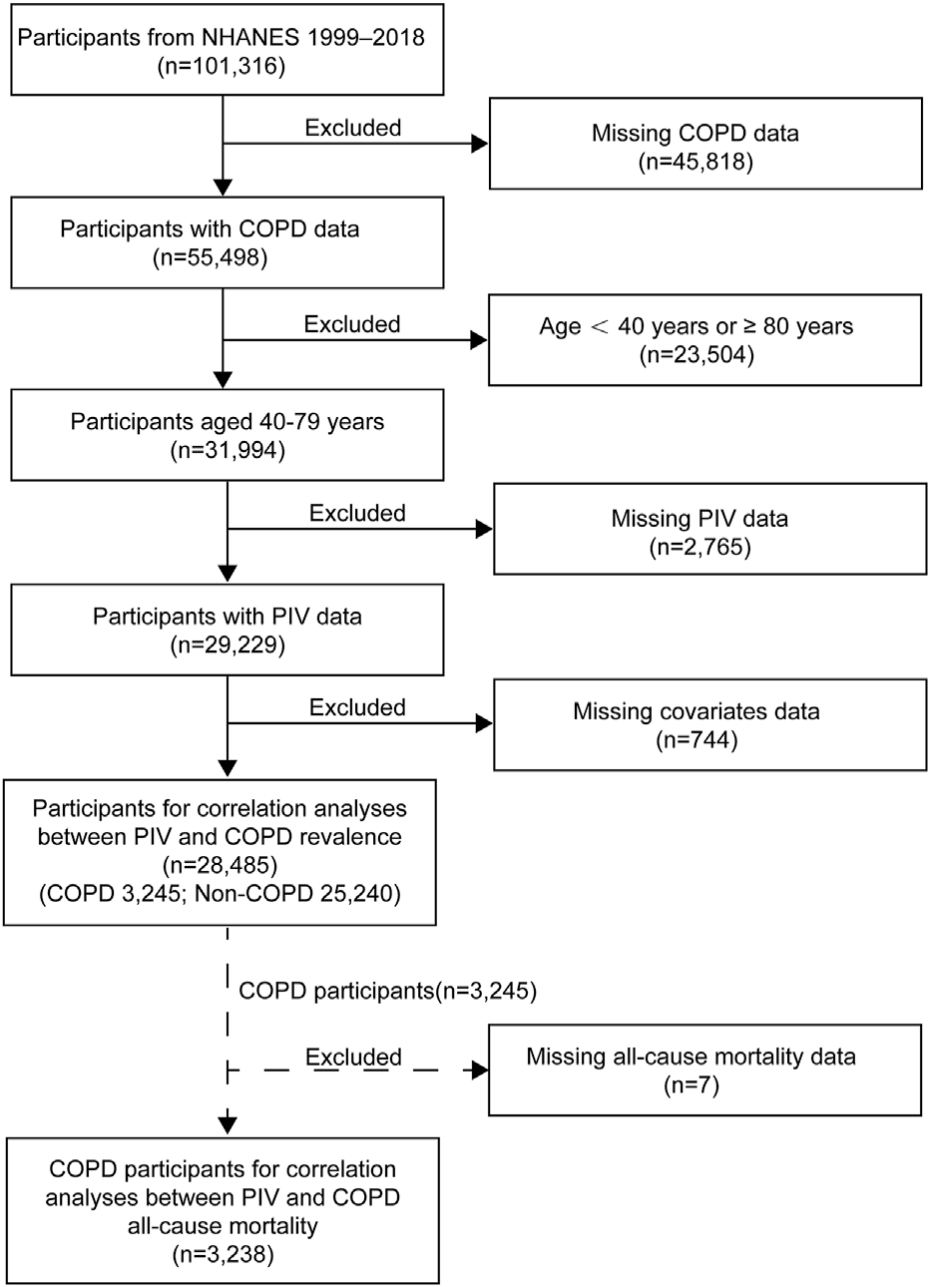
Flowchart of participant selection for this study. NHANES, National Health and Nutrition Examination Survey; COPD, Chronic Obstructive Pulmonary Disease.

Our study initially screened 101,316 participants from NHANES 1999–2018, excluding the following: 1) individuals with missing COPD data; 2) individuals younger than 40 or equal to 80 or older than 80; 3) individuals with missing PIV data; 4) individuals with incomplete covariate data (e.g., BMI, CVD, hypertension, diabetes). Ultimately, a total of 28,485 participants were included in this study (Figure 1).

### 2.2 Assessment of COPD

In accordance with the 2024 Global Initiative for Chronic Obstructive Lung Disease (GOLD) guidelines (GOLD, 2024) and the methodology used in previous studies (Huang et al., 2023a; Liu et al., 2024), we defined COPD as the presence of any of the following: (Christenson et al., 2022) forced expiratory volume in 1 s (FEV1)/forced vital capacity (FVC) <0.7 after bronchodilator inhalation; (Rao et al., 2020) individuals aged 40 or older with a history of smoking or chronic bronchitis who were currently undergoing COPD treatment, including inhaled corticosteroids, mast cell stabilizers, leukotriene modifiers, and selective phosphodiesterase-4 inhibitors; (World Health Organization, 2024) a positive response to any of the questions, “Has a doctor or other health professional ever told you that you had COPD?” “Has a doctor or other health professional ever told you that you had chronic bronchitis?” or “Has a doctor or other health professional ever told you that you had emphysema?”.

### 2.3 Assessment of PIV

The PIV was calculated as neutrophil count (10^9/L) × platelet count (10^9/L) × monocyte count (10^9/L)/lymphocyte count (10^9/L). Log10-PIV represents the logarithmic transformation of PIV with base 10, applied to address the skewed distribution of PIV.

### 2.4 Mortality data collection

We utilized NHANES data from 1999 to 2018 and prospectively correlated it with National Death Index (NDI) mortality data, with follow-up extending December 31, 2019. The primary outcome assessed in this study was all-cause mortality, defined as deaths from any cause.

### 2.5 Covariates

Based on the published studies, we selected several significant potential covariates that might influence the results, including age, sex, race, marriage, educational attainment, smoking status, body mass index (BMI, kg/m2), diabetes, hypertension, and cardiovascular disease (CVD). Age, sex, race, marriage, and educational attainment were obtained from self-reports by the participants. Smoking status was categorized into three groups: former, current, and never smokers. Former smokers were defined as individuals who had smoked at least 100 cigarettes in their lifetime but were not currently smoking. Current smokers were those who had smoked at least 100 cigarettes in their lifetime and were currently smoking either daily or occasionally. Never smokers were defined as individuals who had smoked no more than 100 cigarettes in their lifetime. BMI was grouped as follows: underweight (<18.5 kg/m2), normal (18.5–24.9 kg/m2), overweight (25–29.9 kg/m2), and obese (≥30 kg/m2). Diabetes was diagnosed based on a self-reported physician diagnosis or the application of diabetes medications. Hypertension was defined by a systolic blood pressure ≥130 mmHg, a diastolic blood pressure ≥80 mmHg, self-reported hypertension, or the use of antihypertensive medications. CVD was identified based on an affirmative response to the question, “Has a doctor or other health professional ever told you that you had congestive heart failure (CHF), coronary heart disease (CHD), angina pectoris, heart attack, or stroke?”.

### 2.6 Statistical analyses

All statistical analyses used appropriate sampling weights in accordance with the NHANES Analytic Guidelines to ensure that the sample data accurately represent the entire U.S. population, given the complex multistage probability sampling design. Continuous variables were presented as means (standard error), while categorical variables were expressed as counts (percentages). Due to the skewed distribution of PIV, it was logarithmically transformed for use in regression and subgroup analyses. We applied the weighted Student's t test for continuous variables and the chi-squared test for categorical variables.

Multiple logistic regression analyses were used to examine the associations between PIV and COPD prevalence. Three multivariate-adjusted models were constructed: the crude model without adjusting for any covariates, model 1 adjusting for age, sex, race, marriage, educational attainment, smoking status, and BMI, and model 2 adjusting for all covariates. The results were reported as odds ratios (ORs) and 95% confidence intervals (CIs).

Multiple COX regression analyses and Kaplan-Meier survival curves were utilized to assess the relationships between the PIV and all-cause mortality in COPD participants. Three models were similarly developed following the previous section. The results were indicated as hazard ratios (HRs) and 95% CIs.

We used restricted cubic spline (RCS) analyses to explore the potential nonlinear relationships between PIV and both COPD prevalence and all-cause mortality. Additionally, two-piecewise linear regression analysis was applied to investigate the association between PIV and COPD in more detail. In addition, subgroup analyses were conducted to evaluate the consistency of these relationships across subgroups, and interaction tests were performed to identify the interaction effects among covariates. Furthermore, data from COPD participants with less than 24 months of follow-up were excluded for sensitivity analyses to assess the robustness of the results regarding the relationship between PIV and all-cause mortality in COPD.

All data were analyzed using R software (version 4.3.2). A two-sided p-value <0.05 was considered statistically significant.

## 3 Results

### 3.1 Basic characteristics of participants

This study encompassed a total of 28,485 participants with a mean age of 55.80 years, comprising 14,007 males (47.80%) and 14,478 females (52.20%), including 3,245 COPD participants and 25,240 non-COPD participants. COPD participants may be older than non-COPD participants. Additionally, the two groups differed significantly in sex, race, marriage, educational attainment, smoking status, BMI, diabetes, hypertension, and CVD. Furthermore, the PIV of the COPD group was significantly higher than that of the non-COPD group (all p < 0.0001) (Table 1).

**TABLE 1:** Baseline characteristics of all participants by incident COPD.

Characteristics	Overall (*n* = 28,485)	Non-COPD (*n* = 25,240)	COPD (*n* = 3,245)	P-value
Age (years), n (%)				**<0.0001**
40–49	8,136 (33.68)	7,516 (35.07)	620 (23.22)	
50–59	7,310 (30.88)	6,496 (31.01)	814 (29.94)	
60–69	7,922 (21.99)	6,913 (21.17)	1,009 (28.10)	
70–79	5,117 (13.45)	4,315 (12.74)	802 (18.74)	
Sex, n (%)				**< 0.0001**
Male	14,007 (47.80)	12,538 (48.65)	1,469 (41.39)	
Female	14,478 (52.20)	12,702 (51.35)	1776 (58.61)	
Race, n (%)				**< 0.0001**
Non-Hispanic White	12,435 (72.59)	10,505 (71.56)	1930 (80.36)	
Non-Hispanic Black	6,109 (10.10)	5,479 (10.34)	630 (8.30)	
Mexican American	4,909 (6.15)	4,645 (6.65)	264 (2.40)	
Others	5,032 (11.16)	4,611 (11.45)	421 (8.94)	
Marriage, n (%)				**< 0.0001**
Married/Living with partner	18,508 (70.04)	16,706 (71.08)	1802 (62.19)	
Widowed/Divorced/Separated	7,666 (22.97)	6,484 (21.87)	1,182 (31.23)	
Never married	2,311 (7.00)	2050 (7.05)	261 (6.58)	
Educational attainment, n (%)				**< 0.0001**
>High school	13,781 (58.68)	12,305 (59.59)	1,476 (51.79)	
High school	6,492 (24.15)	5,694 (23.79)	798 (26.80)	
< High school	8,212 (17.17)	7,241 (16.61)	971 (21.41)	
Smoking status, n (%)				**< 0.0001**
Former	8,426 (30.05)	7,146 (28.92)	1,280 (38.68)	
Current	5,724 (19.46)	4,583 (17.36)	1,141 (35.25)	
Never	14,318 (50.46)	13,494 (53.73)	824 (26.07)	
BMI (kg/m^2^), n (%)				**< 0.0001**
Underweight (<18.5)	319 (1.15)	244 (0.99)	75 (2.38)	
Normal (≥18.5,<24.9)	6,652 (24.64)	5,943 (25.00)	709 (21.91)	
Overweight (≥25, <29.9)	10,068 (35.21)	9,110 (35.89)	958 (30.13)	
Obese (≥30)	11,446 (39.00)	9,943 (38.12)	1,503 (45.59)	
Diabetes, n (%)				**< 0.0001**
Yes	5,180 (13.50)	4,374 (12.62)	806 (20.06)	
No	23,305 (86.50)	20,866 (87.38)	2,439 (79.94)	
Hypertension, n (%)				**< 0.0001**
Yes	15,261 (48.07)	13,179 (46.64)	2082 (58.77)	
No	13,224 (51.93)	12,061 (53.36)	1,163 (41.23)	
CVD, n (%)				**< 0.0001**
Yes	3,952 (11.53)	3,013 (9.69)	939 (25.33)	
No	24,533 (88.47)	22,227 (90.31)	2,306 (74.67)	
PIV	324.70 (2.55)	316.89 (2.58)	383.33 (6.49)	**< 0.0001**

Continuous variables were presented as means (standard error). Categorical variables were expressed as counts (percentages).

COPD, chronic obstructive pulmonary disease; PIV, pan-immune-inflammation value; BMI, body mass index; CVD, cardiovascular disease.

The bold values in the table mean statistically significant differences.

### 3.2 Associations between PIV and COPD prevalence

Among the 23,844 participants included, logistic regression analyses were applied to explore the relationship between PIV and the COPD prevalence, as detailed in Table 2. The results indicated a positive correlation between Log10-PIV and COPD, which remained statistically significant in the crude model (OR = 2.52, 95% CI: 2.13–2.99, p < 0.0001), model 1 (OR = 1.77, 95% CI: 1.48–2.12, p < 0.0001), and model 2 (OR = 1.67, 95% CI: 1.39–2.01, p < 0.0001). When Log10-PIV was transformed into a categorical variable by tertiles, the first tertile group (Q1, 0.07≤Log10-PIV≤2.27, n = 9,505), the second tertile group (Q2, 2.27<Log10-PIV≤2.51, n = 9,506), and the third tertile group (Q3, 2.51≤Log10-PIV≤4.36, n = 9,476) were analyzed. The Q2 group (OR = 1.24, 95% CI: 1.10–1.41, p < 0.0001) and Q3 group (OR = 1.79, 95% CI: 1.59–2.03, p < 0.0001) exhibited higher risks of COPD in the crude model. In the model adjusting for selected covariates (model 1), the Q3 group (OR = 1.41, 95% CI: 1.25–1.61, p < 0.0001) had an increased risk of COPD. Similarly, in the model adjusting for all covariates (model 2), only the Q3 group (OR = 1.36, 95% CI: 1.19–1.56, p < 0.0001) demonstrated a higher risk of COPD. Across all models, compared to the reference group (Q1), individuals in the highest tertile (Q3) consistently maintained a positive association with COPD.

**TABLE 2:** Association of PIV with COPD prevalence using logistic regression analyses.

	Crude model		Model 1		Model 2	
	OR (95% CI)	P	OR (95% CI)	P	OR (95% CI)	P
Log10-PIV	2.52 (2.13,2.99)	**<0.0001**	1.77 (1.48,2.12)	**<0.0001**	1.67 (1.39,2.01)	**<0.0001**
Q1 [0.07, 2.27]	References		References		References	
Q2 (2.27, 2.51]	1.24 (1.10,1.41)	**<0.001**	1.12 (0.99,1.26)	0.07	1.10 (0.97,1.24)	0.13
Q3 (2.51, 4.36]	1.79 (1.59,2.03)	**<0.0001**	1.41 (1.25,1.61)	**<0.0001**	1.36 (1.19,1.56)	**<0.0001**
p for trend		**<0.0001**		**<0.0001**		**<0.0001**

Crude Model: no covariates were adjusted.

Model 1: age, sex, race, marriage, educational attainment, smoking status, BMI, were adjusted.

Model 2: all covariates were adjusted.

COPD, chronic obstructive pulmonary disease; PIV, pan-immune-inflammation value; OR, odds ratio; 95% CI: 95% confidence interval; Q1-3 respectively represent the groups divided according to the tertiles.

The bold values in the table mean statistically significant differences.

### 3.3 Associations between PIV and COPD all-cause mortality

Among the 3,238 available death data of COPD participants, during a median follow-up period of 94 months, 852 cases (26%) of all-cause mortality occurred. The final cohort of COPD participants was categorized into three groups based on Log10-PIV tertiles: the first tertile group (Q1, 1.09≤Log10-PIV≤2.35, n = 1,080), the second tertile group (Q2, 2.35<Log10-PIV≤2.60, n = 1,075), and the third tertile group (Q3, 2.60≤Log10-PIV≤3.80, n = 1,083). Baseline characteristics of COPD participants according to Log10-PIV tertiles are detailed in Table 3. The results indicated that individuals with higher Log10-PIV were more likely to be non-Hispanic white, have a higher likelihood of smoking, have a higher tendency towards obesity, and have a greater likelihood with CVD (all p < 0.05).

**TABLE 3:** Baseline characteristics of COPD participants by Log10-PIV tertiles.

Characteristics	Overall (*n* = 3,238)	Q1 (*n* = 1,080)	Q2 (*n* = 1,079)	Q3 (*n* = 1,079)	P-value
Age(years), n (%)					0.11
40–49	618 (23.23)	204 (22.44)	220 (24.97)	194 (22.10)	
50–59	812 (29.92)	284 (31.47)	275 (29.64)	253 (28.79)	
60–69	1,006 (28.09)	372 (30.00)	326 (27.69)	308 (26.76)	
70–79	802 (18.77)	220 (16.09)	258 (17.69)	324 (22.36)	
Sex, n (%)					0.2
Male	1,465 (41.37)	451 (40.91)	467 (39.27)	547 (44.04)	
Female	1773 (58.63)	629 (59.09)	612 (60.73)	532 (55.96)	
Race, n (%)					**< 0.0001**
Non-Hispanic White	1928 (80.42)	503 (72.08)	692 (83.88)	733 (84.34)	
Non-Hispanic Black	629 (8.31)	353 (15.18)	148 (5.66)	128 (4.84)	
Mexican American	263 (2.40)	68 (2.22)	104 (2.54)	91 (2.41)	
Others	418 (8.88)	156 (10.52)	135 (7.92)	127 (8.41)	
Marriage, n (%)					0.05
Married/Living with partner	1796 (62.14)	599 (63.85)	616 (64.50)	581 (58.07)	
Widowed/Divorced/Separated	1,181 (31.27)	376 (28.97)	397 (30.16)	408 (34.55)	
Never married	261 (6.59)	105 (7.17)	66 (5.33)	90 (7.38)	
Educational attainment, n (%)					0.22
>High school	1,472 (51.81)	519 (54.65)	489 (52.44)	464 (48.55)	
High school	796 (26.76)	264 (26.11)	260 (26.15)	272 (28.00)	
< High school	970 (21.43)	297 (19.24)	330 (21.41)	343 (23.45)	
Smoking status, n (%)					**< 0.0001**
Former	1,276 (38.62)	386 (35.58)	449 (42.11)	441 (37.69)	
Current	1,140 (35.29)	348 (32.47)	353 (31.40)	439 (42.01)	
Never	822 (26.08)	346 (31.95)	277 (26.48)	199 (20.30)	
BMI (kg/m^2^), n (%)					**0.01**
Underweight (<18.5)	75 (2.38)	20 (1.21)	25 (2.51)	30 (3.32)	
Normal (≥18.5,<24.9)	707 (21.91)	243 (23.62)	217 (19.05)	247 (23.41)	
Overweight (≥25, <29.9)	956 (30.12)	342 (33.98)	312 (29.12)	302 (27.66)	
Obese (≥30)	1,500 (45.58)	475 (41.19)	525 (49.32)	500 (45.61)	
Diabetes, n (%)					0.88
Yes	806 (20.09)	265 (19.55)	257 (20.61)	284 (20.02)	
No	2,432 (79.91)	815 (80.45)	822 (79.39)	795 (79.98)	
Hypertension, n (%)					0.27
Yes	2078 (58.76)	682 (55.88)	682 (59.72)	714 (60.38)	
No	1,160 (41.24)	398 (44.12)	397 (40.28)	365 (39.62)	
CVD, n (%)					**0.01**
Yes	939 (25.37)	281 (23.29)	293 (23.38)	365 (29.38)	
No	2,299 (74.63)	799 (76.71)	786 (76.62)	714 (70.62)	

Continuous variables were presented as means (standard error). Categorical variables were expressed as counts (percentages).

COPD, chronic obstructive pulmonary disease; PIV, pan-immune-inflammation value; BMI, body mass index; CVD, cardiovascular disease.

The bold values in the table mean statistically significant differences.

**FIGURE 2:**
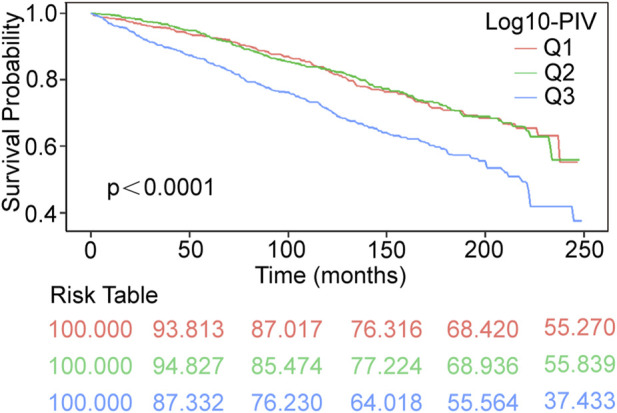
Kaplan–Meier survival curves of COPD all-cause mortality during 249 months follow–up duration. COPD, Chronic Obstructive Pulmonary Disease; PIV, pan-immune-inflammation value; Q1-3 respectively represent the groups divided according to the tertiles; Q1 [1.09, 2.35]; Q2 (2.35, 2.60]; Q3 (2.60, 3.80].

We generated Kaplan-Meier survival curves with accompanying risk tables, using weighted survival rates at a follow-up duration of 249 months as the endpoint. In the Kaplan-Meier survival curves, the Q3 group exhibited the highest all-cause mortality (p < 0.0001, Figure 2). COX regression analyses showed a significant positive correlation between Log10-PIV and the COPD all-cause mortality in the crude model (HR = 2.86, 95% CI: 1.94–4.21, p < 0.0001), model 1 (HR = 2.12, 95% CI: 1.49–3.03, p < 0.0001), and model 2 (HR = 2.04, 95% CI: 1.41–2.95, p < 0.001). In all models, compared to the Q1 group, the Q3 group had a higher risk of all-cause mortality (crude model: HR = 1.78, 95% CI: 1.40–2.27, p < 0.0001; model 1: HR = 1.54, 95% CI: 1.22–1.94, p < 0.001; model 2: HR = 1.49, 95% CI: 1.17–1.89, p = 0.001) (Table 4).

**TABLE 4:** Association of PIV with COPD all-cause mortality using COX regression analyses.

	Crude model		Model 1		Model 2	
	HR (95% CI)	P	HR (95% CI)	P	HR (95% CI)	P
Log10-PIV	2.86 (1.94,4.21)	**<0.0001**	2.12 (1.49,3.03)	**<0.0001**	2.04 (1.41,2.95)	**<0.001**
Q1 [1.09, 2.35]	References		References		References	
Q2 (2.35, 2.60]	1.00 (0.81,1.23)	0.98	0.95 (0.77,1.18)	0.65	0.95 (0.76,1.18)	0.62
Q3 (2.60, 3.80]	1.78 (1.40,2.27)	**<0.0001**	1.54 (1.22,1.94)	**<0.001**	1.49 (1.17,1.89)	**0.001**
p for trend		**<0.0001**		**<0.0001**		**<0.001**

Crude Model: no covariates were adjusted.

Model 1: age, sex, race, marriage, educational attainment, smoking status, BMI, were adjusted.

Model 2: all covariates were adjusted.

COPD, chronic obstructive pulmonary disease; PIV, pan-immune-inflammation value; HR, hazard ratio; 95% CI: 95% confidence interval; Q1-3 respectively represent the groups divided according to the tertiles.

The bold values in the table mean statistically significant differences.

### 3.4 Identification of nonlinear relationship

**FIGURE 3:**
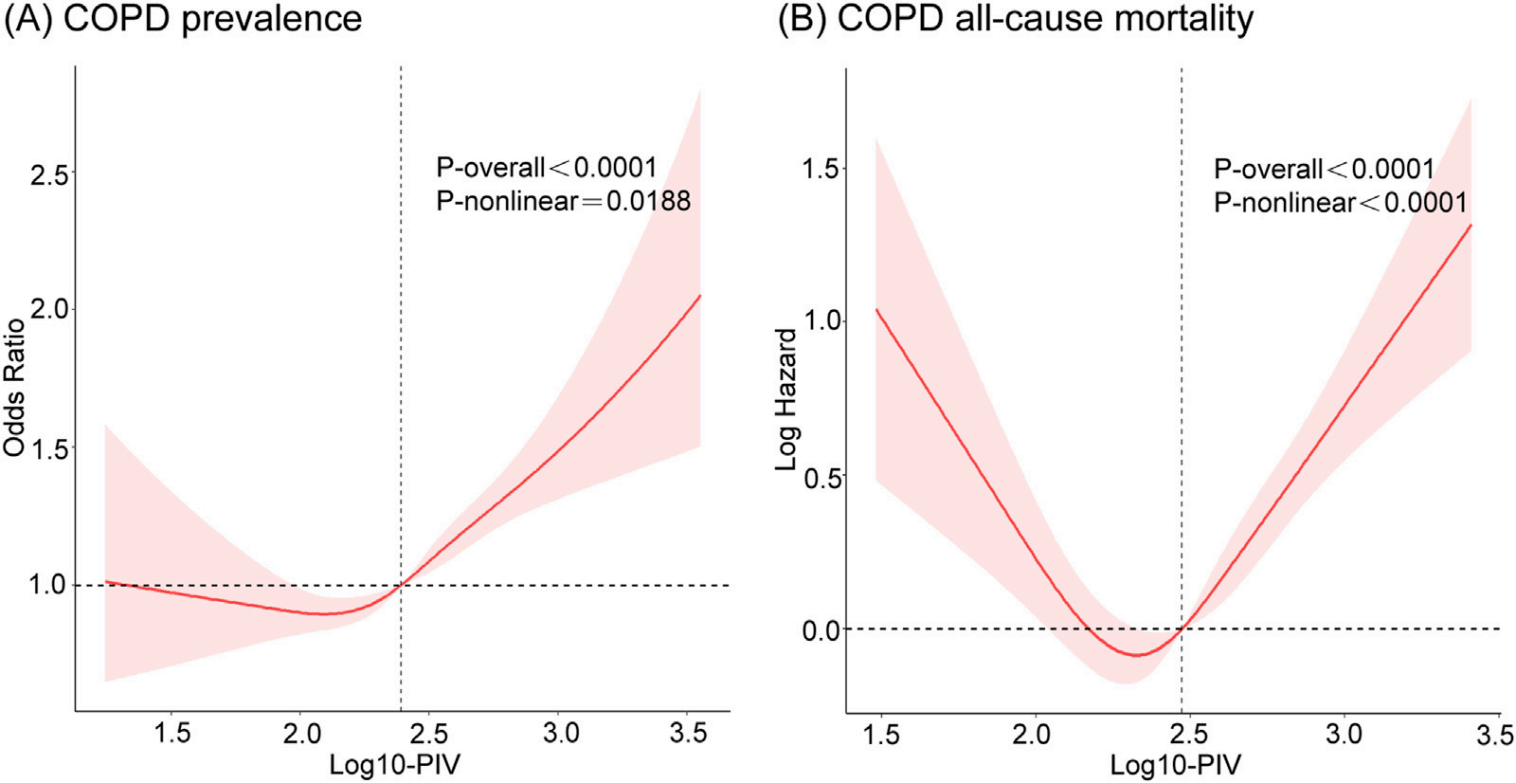
The RCS curves of the associations of PIV with COPD prevalence and COPD all-cause mortality after adjusting all covariates. **(A)** COPD prevalence; **(B)** COPD all-cause mortality. COPD, Chronic Obstructive Pulmonary Disease; PIV, pan-immune-inflammation value.

To identify the potential nonlinear relationships between PIV and COPD, RCS analyses were applied after adjusting for all covariates. The results are presented in Figure 3. Log10-PIV showed a nonlinear correlation with COPD, exhibiting a J-shaped curve for COPD prevalence (p for nonlinear <0.05) and a U-shaped curve for COPD all-cause mortality (p for nonlinear <0.0001). The RCS curves exhibited distinct inflection points, prompting the execution of segmented linear regression analyses, as detailed in Table 5. The results revealed a significant inflection point (K = 2.24) between Log10-PIV and COPD prevalence. When Log10-PIV was below 2.24, the association between Log10-PIV and COPD prevalence was not statistically significant (OR = 0.86, 95% CI: 0.45–1.64, p = 0.65), whereas it was significantly positive when Log10-PIV exceeded 2.24 (OR = 2.00, 95% CI: 1.57–2.55, p < 0.0001). A distinct inflection point was also identified for Log10-PIV and COPD all-cause mortality (K = 2.38). On the left side of the inflection point, Log10-PIV had a strong negative correlation with all-cause mortality (HR = 0.23, 95% CI: 0.12–0.44, p < 0.0001). On the right side of the inflection point, there was a significant positive correlation between Log10-PIV and all-cause mortality (HR = 4.12, 95% CI: 2.62–6.48, p < 0.0001). These findings indicate that PIV levels have varying effects on COPD.

**TABLE 5:** Two-piecewise linear regression analyses of PIV and COPD prevalence and COPD all-cause mortality after adjusting all covariates.

Log10-PIV	Adjusted OR (95% CI) P	Adjusted HR (95%CI) P
Fitting by the standard linear model	1.64 (1.36, 1.97) <0.0001	2.04 (1.41, 2.95) <0.001
Fitting by the two-piecewise linear model		
Inflection point(K)	2.24	2.38
<K	0.86 (0.45, 1.64) 0.65	0.23 (0.12, 0.44) <0.0001
≥K	2.00 (1.57, 2.55) <0.0001	4.12 (2.62, 6.48) <0.0001
Log likehood ratio test	P < 0.001	P < 0.001

The results of PIV, and COPD prevalence were expressed as Adjusted OR (95% CI).

The results of PIV, and COPD all-cause mortality were expressed as Adjusted HR (95% CI).

COPD, chronic obstructive pulmonary disease; PIV, pan-immune-inflammation value; OR, odds ratio; HR, hazard ratio; 95% CI: 95% confidence interval.

### 3.5 Subgroup analyses and sensitivity analyses

**FIGURE 4:**
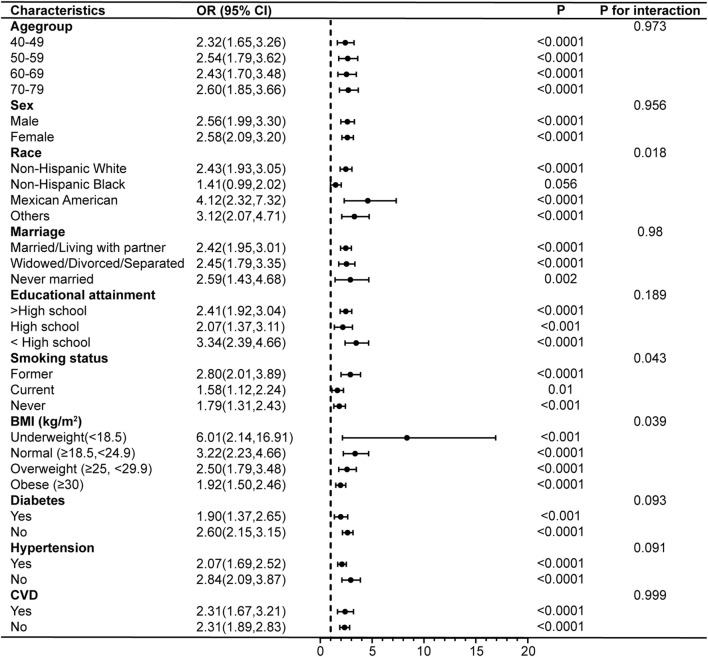
Subgroup analyses of the associations between PIV and COPD prevalence. COPD, Chronic Obstructive Pulmonary Disease; PIV, pan-immune-inflammation value; BMI, body mass index; CVD, cardiovascular disease; OR, odds ratio; 95% CI: 95% confidence interval.

**FIGURE 5:**
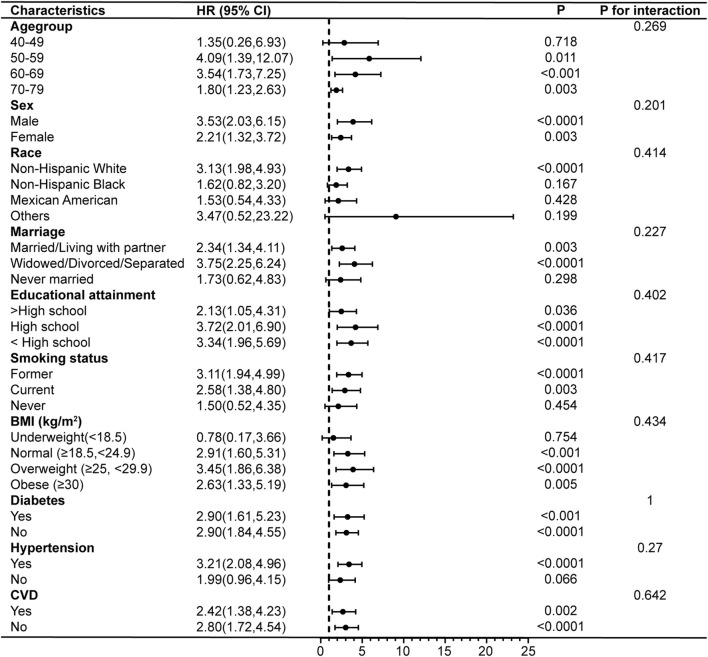
Subgroup analyses of the associations between PIV and COPD all-cause mortality. COPD, Chronic Obstructive Pulmonary Disease; PIV, pan-immune-inflammation value; BMI, body mass index; CVD, cardiovascular disease; HR, hazard ratio; 95% CI: 95% confidence interval.

Subgroup analyses and interaction tests were conducted after adjusting for all covariates to further validate the consistency of the association between PIV and COPD and to identify potential differences in specific subgroups. The results showed that the association between PIV and COPD prevalence remained unchanged after stratifying the participants by age, sex, marriage, educational attainment, diabetes, hypertension, and CVD (p for interaction >0.05). However, there were significant interactions between PIV and race, smoking status, and BMI. PIV and the risk of COPD prevalence were not strongly associated among non-Hispanic Black (OR = 1.41, 95% CI: 0.99–2.02), but showed stronger associations among Mexican Americans (OR = 4.12, 95% CI: 2.32–7.32), former smokers (OR = 2.80, 95% CI: 2.01–3.89), and those with a BMI classified as underweight (OR = 6.01, 95% CI: 2.14–16.91) (Figure 4). In addition, the results demonstrated no interaction in the association between PIV and COPD all-cause mortality across all subgroups (all p for interaction >0.05), indicating consistent results across subgroups (Figure 5).

Sensitivity analyses were performed by excluding individuals with follow-up times of less than 24 months (Supplementary Table S1), and the results of COX regression analyses remained consistent, thereby validating the robustness of our findings.

## 4 Discussion

To the best of our knowledge, this study is the first to elucidate the associations between PIV and COPD prevalence and all-cause mortality in a representative national sample. We included eligible participants from 1999 to 2018 in the NHANES database, and the study yielded the following main findings: 1) PIV was positively correlated with both COPD prevalence and all-cause mortality, with this relationship remaining significant in fully adjusted models. 2) PIV exhibited a J-shaped correlation with COPD prevalence and a U-shaped correlation with all-cause mortality. This suggests that an increase in PIV is significantly associated with higher COPD prevalence within certain ranges, whereas COPD all-cause mortality initially decreased and subsequently increased with rising PIV levels. 3) Subgroup analyses demonstrated that the association between PIV and COPD prevalence was influenced by race, smoking status, and BMI. This association was not significant among non-Hispanic Black individuals but was stronger in Mexican Americans, former smokers, and those with underweight BMI. However, the association between PIV and COPD all-cause mortality remained consistent across all subgroups and was unaffected by common demographic factors, lifestyle, and underlying conditions. These results could facilitate the clinical development of PIV as a more comprehensive composite indicator for assessing COPD diagnosis, prognosis, and the identification of high-risk COPD populations.

PIV combines four major peripheral blood immune cell types: neutrophils, platelets, monocytes, and lymphocytes, and is considered a novel and more comprehensive indicator of systemic inflammation. Previous research has primarily focused on cancer patients. For instance, a systematic review and meta-analysis of 15 studies involving 4,942 patients demonstrated that PIV serves as a prognostic biomarker for both overall survival and progression-free survival in cancer patients (Guven et al., 2022). Similar results were reported in another systematic review and meta-analysis focusing on colon cancer (Yang et al., 2022). In pulmonary tumors, PIV has served as a predictive biomarker for non-small cell lung cancer (Putzu et al., 2018) and as an indicator of pathological complete response and clinical prognosis in patients undergoing neoadjuvant immunotherapy for non-small cell lung cancer (Zhai et al., 2023). Recently, the prognostic value of PIV in non-cancerous diseases has been recognized. For example, PIV has been regarded as a prognostic indicator for outcomes in patients with ST-segment elevation myocardial infarction and non-traumatic subarachnoid hemorrhage (Murat et al., 2023; Huang et al., 2023b). In pulmonary diseases, PIV has been viewed both as an indicator for assessing the severity of COVID-19 and the necessity of ICU admission (Hamad et al., 2022), and as a prognostic indicator for adverse outcomes in patients with idiopathic pulmonary fibrosis (Zinellu et al., 2020). Recently, a study indicated that AISI upon admission serves as a reliable predictor of mortality in COVID-19 patients with COPD, with higher AISI values correlating with lower survival rates (Hosseninia et al., 2023). In our study, COPD participants exhibited higher PIV levels compared to non-COPD participants. The highest tertile of PIV was associated with the highest prevalence and all-cause mortality. Logistic regression and COX regression revealed that high PIV levels are independent risk factors for both COPD prevalence and all-cause mortality. These findings are consistent with previous studies, further confirming the clinical utility of PIV in COPD.

Our research confirmed a positive correlation between PIV and the risk of COPD prevalence and all-cause mortality. However, the underlying pathological and physiological mechanisms remain unclear. Current consensus suggests that the pathogenesis of COPD primarily involves inflammatory mechanisms, protease-antiprotease imbalance, and oxidative stress from various sources, collectively contributing to the development of small airway and emphysematous lesions, which result in the characteristic persistent airflow limitation of COPD (Mirza et al., 2018). From the perspective of PIV, neutrophils are the most abundant cells in circulation and are the first to be recruited to the site of inflammation (Sadiku et al., 2021). The activation and degranulation of neutrophils release various destructive enzymes, including neutrophil elastase (NE), matrix metalloproteinase (MMP), proteinase 3 (PR3), and cathepsin G (Cat G), leading to lung tissue damage (Chalmers et al., 2023; Jasper et al., 2019). NE stimulates toll-like receptors (TLRs) on epithelial cells, resulting in the upregulation and release of cytokines, increased production and secretion of airway mucoproteins, excessive mucus secretion, and subsequent airway obstruction (Voynow et al., 2020; Hao et al., 2020). Additionally, NE upregulates neutrophil chemotactic factors, such as interleukin (IL)-8, thereby amplifying inflammation (Clancy et al., 2018). Compared to NE, PR3 may experience fewer pulmonary inhibitory effects and have a broader radius of activity (Crisford et al., 2018). Platelets are not only important in hemostasis and thrombosis but also play a crucial role in immune and inflammatory regulation (Mallah et al., 2020). Activated platelets produce and release inflammatory mediators, such as IL-1β, recruiting inflammatory cells and driving oxidative stress and chronic inflammation in COPD (Koenen, 2016). Studies have shown that impaired repair and remodeling following lung injury are closely associated with COPD, where hemostatic disorders and chronic inflammation play major roles (Agustí and Hogg, 2019). In COPD patients, however, platelet activation disorders leading to impaired hemostasis hinder lung tissue repair (Liu et al., 2020), thereby promoting the development of COPD. Platelets can also mediate the formation of pulmonary microthrombi. Red blood cells in COPD patients have been observed to undergo deformation, facilitating the translocation of platelets to the vascular wall, which results in adhesion, aggregation, and activation on the vascular wall (Zouaoui Boudjeltia et al., 2021). Furthermore, hypoxia stress and elevated expression of hypoxia-inducible factor (HIF) due to hypoxemia in COPD can stimulate platelets to synthesize plasminogen activator inhibitor (PAI)-1, inducing a prothrombotic state associated with hypoxia (Chaurasia et al., 2019), which further promotes the development of COPD. Monocytes are recruited to the lungs and contribute to the macrophage pool during inflammation, increasing the macrophage population in the airways of COPD patients (Barnes, 2017). Macrophages secrete numerous inflammatory mediators, including IL-1β, tumor necrosis factor-α (TNF-α), IL-8, MMPs, and reactive oxygen species (ROS), which directly or indirectly affect airway structural cells and participate in airway remodeling (Wang et al., 2018a). Studies have found that monocyte-derived macrophages (MDMs) in COPD exhibit defects in phagocytosis and engulfment, leading to excessive cellular inflammation, an ineffective immune response, and ultimately lung injury, potentially associating with the frequency of acute exacerbations of COPD (Bewley et al., 2017; Ryan et al., 2023). Lymphocytes contribute to lung tissue damage in COPD patients (Wang et al., 2018a). Subtypes of CD4^+^ T cells, specifically Th17 cells, secrete IL-17, which stimulates airway epithelial cells to produce chemokines and other mediators, recruiting and activating neutrophils and macrophages (Pridgeon et al., 2011). Activated CD8^+^ T cells release cytotoxic mediators, such as perforin, which damage lung tissue structure and accelerate the progression of emphysema (Kobayashi et al., 2013). In addition, CD8^+^ T cells produce various inflammatory mediators, including interferon-γ (IFN-γ), TNF-α, and IL-4, which may be linked to exacerbations of COPD (Barczyk et al., 2006). These studies demonstrate the intricate biological relationship between neutrophils, platelets, monocytes, and lymphocytes and the pathogenesis of COPD. From an epidemiological perspective, our findings reveal a clear connection between PIV and COPD.

Using RCS analyses, we elucidated the nonlinear relationships between PIV and COPD prevalence and all-cause mortality. The prevalence curve exhibited a J-shaped correlation, and the all-cause mortality curve showed a U-shaped correlation, suggesting that elevated PIV increased the risk of COPD prevalence, while both elevated and reduced PIV increased the risk of all-cause mortality in COPD patients. Currently, there is no precise research elucidating the reasons for these outcomes. We speculated that this might be due to the fact that a low PIV level reflects an individual’s immune deficiency state, whereas a high PIV level indicates an excessive state. Both conditions might lead to immune imbalance in COPD patients and subsequent increase the risk of mortality. This suggests a potential beneficial effect of PIV within a specific range in reducing the risk of COPD prevalence and all-cause mortality.

After conducting subgroup analyses that adjusted for potential confounding factors, the associations between PIV and all-cause mortality in COPD patients remained consistent across different subgroups. However, our results revealed specific differences in the associations between PIV and COPD prevalence among various populations. PIV did not affect the prevalence risk in non-Hispanic Black individuals but showed a stronger correlation in Mexican Americans who were former smokers and had a BMI classified as underweight. These suggest that these individuals might constitute a high-risk population for COPD and require more intensive prevention strategies. Previous studies consistently demonstrate an increased risk of COPD associated with smoking and underweight (Wang et al., 2018b; Li et al., 2020), which aligns with our findings. Research indicates that smoking is a significant etiological factor for COPD, leading to increased production of reactive oxygen species and persistent inflammation even after smoking cessation (Rao et al., 2020). The increased risk of COPD associated with underweight may be related to malnutrition and muscle weakness (Benz et al., 2019; Shaheen and Barker, 1994). Although the interaction mechanisms between race and PIV remain unclear, a recent cohort study showed a lower likelihood of COPD in Black individuals compared to other races (Ejike et al., 2021), which is consistent with our results. However, this study also reported that Black individuals were associated with higher disease severity and acute exacerbation risks (Ejike et al., 2021), suggesting the need for further research to explore potential influencing factors such as race-specific genetics, physical function, and individual socioeconomic status.

Our study has several key strengths. Firstly, this study represents the inaugural investigation into the association between PIV and both COPD prevalence and all-cause mortality, utilizing a nationally representative sample from the United States. This approach enhances the generalizability and applicability of our findings. Secondly, we controlled for numerous potential confounding factors, including demographic information, lifestyle, and comorbidities, thereby enhancing the reliability and representativeness of our results. Thirdly, RCS analyses were applied to identify potential nonlinear relationships and cut-off values, while segmented linear regression analyses were used to more precisely describe the relationship between PIV and COPD. Finally, subgroup analyses with interaction verification and sensitivity analyses validated the robustness of our results and highlighted potential heterogeneity.

However, this study also has limitations. Firstly, our study population comprised individuals aged 40–79 from the United States. This was due to the fact that COPD patients are predominantly over the age of 40 (5). In addition, it was to exclude deaths caused by various factors, such as aging, among individuals older than 80. This may limit the generalizability of the findings to other populations. Secondly, some participants defined as having COPD were prescribed medication, which may have influenced PIV levels. Future research employing more in-depth and refined methodologies is necessary. Thirdly, although we adjusted for various confounding factors, other potential confounders could not be entirely accounted for because of many constraints, including space limitations. Fourthly, we are unable to infer causality between PIV and COPD because of the cross-sectional design of NHANES; therefore, further prospective studies and randomized trials are required to confirm these associations. Finally, the correlation between PIV and COPD prevalence is influenced by race, smoking status, and BMI, requiring further research into these factors to explore potential mechanisms and enhance precision medicine.

In summary, this study found that elevated PIV levels were associated with increased COPD prevalence, particularly among Mexican Americans, former smokers, and individuals with a BMI classified as underweight. This finding may facilitate the identification of at-risk populations and guide more effective management strategies. Additionally, the study revealed that COPD patients with either elevated or reduced PIV levels faced a higher risk of all-cause mortality. These findings provide valuable clues for targeted interventions aimed at reducing COPD prevalence and all-cause mortality and emphasize the potential of PIV as a biomarker.

## 5 Conclusion

Elevated PIV levels are associated with increased COPD prevalence. COPD patients with either elevated or reduced PIV levels experience higher all-cause mortality. Further large-scale, longitudinal studies are required to corroborate these findings.

## Data Availability

Publicly available datasets were analyzed in this study. This data can be found here: https://www.cdc.gov/nchs/nhanes/index.htm.
