# Supplementary material for: The association between pan-immune-inflammation value and chronic obstructive pulmonary disease: data from NHANES 1999–2018
Source: Front Physiol. 2024 Oct 7;15:1440264. doi: 10.3389/fphys.2024.1440264 (PMC11491374; doi:10.3389/fphys.2024.1440264)
Supplement: Supplementary file 1 [file Table1.DOCX]

Supplementary Material

Supplementary Table 1. Sensitivity analyses by excluding individuals with a follow-up time less than 24 months.

|  | Crude Model | | Model 1 | | Model 2 | |
| --- | --- | --- | --- | --- | --- | --- |
|  | HR(95% CI) | P | HR(95% CI) | P | HR(95% CI) | P |
| Log10-PIV | 2.46(1.65,3.67) | **<0.0001** | 1.85(1.30,2.64) | **<0.001** | 1.76(1.22,2.56) | **0.003** |

Crude Model: no covariates were adjusted.

Model 1: age, sex, race, marriage, educational attainment, smoking status, BMI were adjusted.

Model 2: all covariates were adjusted.

COPD, Chronic Obstructive Pulmonary Disease; HR: hazard ratio; 95% CI: 95% confidence interval; Q1-3 respectively represent the groups divided according to the tertiles.
